# The algorithmic palliative medicine clinic: Prognosis, temporality, and the burden of anticipation

**DOI:** 10.1017/S1478951526103198

**Published:** 2026-09-01

**Authors:** Abel García Abejas

**Affiliations:** 1 Universidade da Beira Interior Faculdade de Ciencias da Saude, Covilhã, Portugal; 2 Faculty of Medicine, Universidad Francisco de Vitoria, Madrid, Spain

## Introduction

Medicine is increasingly a discipline of anticipation. In palliative medicine, this transformation is most visible in the proliferation of predictive analytics, machine learning systems, electronic patient-reported outcome measures (ePROMs), wearable technologies, and algorithmic decision-support tools designed to anticipate deterioration, estimate mortality risk, and optimize referral to specialist palliative care (De Panfilis et al. 2023; Wilson et al. 2023). The predictive turn responds to a genuine clinical problem: patients with advanced cancer and chronic progressive illness continue to experience delayed referral, excessive therapeutic escalation, and avoidable suffering before supportive care is adequately integrated (Murray et al. 2005; Neto et al. 2025). Earlier identification of palliative care needs may improve anticipatory planning, facilitate serious illness conversations, and reduce burdensome interventions (Porter et al. 2020). Yet prognosis is never ethically neutral.

Predictive knowledge does not remain confined to the informational domain once it enters clinical life. Prognostic statements reshape emotional horizons, influence family dynamics, reorganize expectations, and modify how remaining time is inhabited. Artificial intelligence intensifies this tension because it alters not only the accuracy of prognostic estimation but also the mode through which prognosis becomes present within care. Predictive systems increasingly identify vulnerability before suffering is subjectively integrated, classify deterioration before decline becomes experientially meaningful, and render future mortality psychologically visible long before patients are emotionally or narratively prepared. In predictive medicine, the future increasingly becomes clinically active before it is existentially inhabited.

This article argues that the ethical problem introduced by predictive AI in palliative medicine is not prediction itself – prognostic reasoning has always been central to medicine – but the risk that probabilistic anticipation acquires disproportionate existential authority over lived experience. Human beings do not inhabit mortality as statistical trajectories. They continue reinterpreting suffering, renegotiating meaning, sustaining relational bonds, and narratively inhabiting finitude even under conditions of severe decline. Life near the end remains temporally and ethically open despite prognostic probability.

To conceptualize this phenomenon, this article proposes the notion of *algorithmic anticipatory burden*: the existential, relational, and temporal weight generated when AI-mediated predictions concerning deterioration or mortality become psychologically, clinically, or institutionally active before the person is ready to inhabit them. The patient no longer lives only with illness, but progressively beneath its anticipation.

This concept differs fundamentally from anxiety, prognostic distress, or ordinary existential uncertainty. It also differs from what Murray described as *prognostic paralysis* – the hesitation of clinicians to initiate palliative care conversations due to prognostic uncertainty (Murray et al. 2005). Algorithmic anticipatory burden emerges not from insufficient prognostic confidence, but from excessive anticipatory authority once probabilistic knowledge becomes clinically and psychologically operative. Drawing on phenomenology, narrative ethics, ethics of care, and philosophy of technology, this article examines how predictive systems transform temporality, relationality, and the lived experience of vulnerability – and proposes a prudential ethics of prediction capable of preserving existential openness under conditions of increasing anticipatory visibility.

## Prognosis and the transformation of clinical temporality

Prognosis has always occupied a central but unstable position within medicine. In its classical sense, prognostic judgment was a prudential and interpretive practice grounded in temporal observation, embodied presence, narrative understanding, and practical wisdom. It functioned not merely as prediction, but as orientation – helping patients inhabit uncertainty while preserving meaning, dignity, and relational continuity. This dimension is particularly important in palliative care, where prognostic judgment directly intervenes in the temporal structure of lived experience, shaping how patients and families understand remaining time, hope, and mortality itself.

Contemporary predictive AI appears to offer a solution to the longstanding problem of prognostic paralysis. Machine learning systems, electronic health record-based models, and algorithmic triage tools promise earlier recognition of deterioration, more accurate mortality estimation, and more timely identification of patients requiring specialist palliative care (De Panfilis et al. 2023; He et al. 2024; Heinzen et al. 2023; Wilson et al. 2023). The ethical appeal is understandable: if predictive systems can identify hidden vulnerability earlier than unaided clinical judgment, they may reduce suffering, prevent burdensome interventions, and create opportunities for more humane care.

However, the transition from prognosis to algorithmic prediction represents more than quantitative improvement in predictive precision. It marks a deeper epistemological and temporal transformation. Classical prognosis is fundamentally interpretive and relational, unfolding through dialogue, uncertainty, longitudinal observation, and narrative contextualization, remaining revisable, situated, and ethically mediated. Algorithmic prediction functions differently: it produces probabilistic outputs through computational correlations extracted from large datasets, rendering the patient through variables, risk scores, and predictive classifications. More significantly, it reorganizes temporality itself.

Classical prognosis generally leaves space for interpretive openness; prognostic estimations coexist with uncertainty and evolving emotional adaptation. The future remains partially indeterminate. Algorithmic prediction, by contrast, progressively transforms future possibility into present visibility. Mortality becomes operationalized through alerts, probabilities, dashboards, and continuously updated risk estimations. The anticipated future acquires psychological and institutional presence before it is existentially integrated. Predictive systems render decline visible before it becomes subjectively meaningful. The patient may become classified as “high risk” or “approaching end of life” months before mortality becomes experientially central. Prediction no longer functions merely as orientation toward possible futures; it begins reorganizing the present itself.

## Algorithmic anticipatory burden

Algorithmic anticipatory burden refers to the existential, relational, temporal, and institutional weight generated when AI-mediated predictions concerning deterioration or mortality become psychologically, clinically, or socially operative before the person is ready to inhabit them. It emerges when probabilistic anticipation ceases to function merely as orientation toward possible futures and progressively reorganizes lived experience around anticipated decline.

The burden does not imply that anticipation is intrinsically pathological or that prognostic reasoning should be avoided. It emerges specifically when predictive systems relocate existence beneath anticipated futures before those futures have become experientially or existentially integrated. Several mechanisms contribute to this process.

### Predictive visibility and temporal activation

Contemporary predictive systems render future deterioration visible before it becomes subjectively meaningful. Machine learning models identify patterns associated with mortality months before patients consciously experience themselves as approaching death (Liu et al. 2023; Strand et al. 2024). Through predictive dashboards, deterioration alerts, and algorithmic triage tools, future vulnerability becomes continuously present within clinical infrastructures. The future ceases to remain merely possible and progressively becomes psychologically operative within the present. Decline is no longer only awaited or feared; it is computationally projected and institutionally activated, generating what may be described as *anticipatory hypervigilance*: patients become increasingly attentive to bodily changes through the lens of anticipated mortality, families reorganize interactions around expected decline, and clinicians become oriented toward projected outcomes rather than the ambiguity of lived experience.

### Risk internalization and probabilistic identity

Predictive medicine classifies persons through probabilistic categories – “high risk,” “frail,” “end-of-life trajectory” – that may be clinically useful yet possess symbolic power. Once repeatedly encountered within medical interactions and family conversations, predictive categories may progressively become incorporated into self-understanding. The patient risks what may be called a *probabilistic identity*: existence increasingly reorganized around projected outcomes rather than present relationships, memory, and ordinary temporality. Ricoeur demonstrates that narrative identity depends upon the capacity to continuously reinterpret oneself through an evolving relationship between past, present, and future (Ricoeur 1992). Predictive classification may threaten this openness when projected futures become excessively stabilized – possibility narrows into expectation, and the patient’s narrative horizon risks becoming colonized by anticipated endings.

### Temporal colonization and the compression of existential space

The deepest dimension of algorithmic anticipatory burden is *temporal colonization*: the gradual occupation of lived time by anticipated future. Under conditions of persistent predictive visibility, the future progressively acquires disproportionate existential density within the present. Remaining time ceases to unfold through open temporality and increasingly becomes reorganized around expected deterioration. Even moments of stability may become psychologically overshadowed. Heidegger’s analysis clarifies the stakes: authentic being-toward-death arises from the individual’s own confrontation with finitude, whereas algorithmic anticipation risks replacing existential openness with computational imminence (Heidegger 1996). Anticipatory burden becomes particularly acute near the end of life because remaining time often possesses heightened symbolic density – reconciliation, ordinary continuity, memory work, relational repair, spiritual struggle – dimensions that require temporal openness and cannot fully unfold within an atmosphere dominated by predictive saturation.

## Temporality, finitude, and narrative existence

The ethical tensions introduced by predictive AI cannot be fully understood without examining temporality itself. Prognostic systems do not merely provide information; they reorganize how time is psychologically inhabited, narratively interpreted, and relationally experienced. In end-of-life care, this is never neutral: serious illness already alters the structure of expectation, memory, embodiment, and identity. Predictive AI intensifies this disruption.

For Heidegger, human existence is fundamentally temporal; persons do not merely exist within chronological succession but project themselves toward possibilities (Heidegger 1996). Mortality constitutes an existential horizon giving urgency, fragility, and singularity to existence. Crucially, authentic anticipation of death differs from externally imposed prediction. Existential anticipation arises from the individual’s confrontation with finitude; algorithmic anticipation may impose mortality as administratively mediated probability. AI-based prognostic systems transform mortality into predictive visibility – the patient encounters death not only through existential awareness but also through computational outputs, mortality scores, and continuously updated risk trajectories. Mortality becomes statistically operationalized, risking transformation from existential horizon into permanent predictive imminence.

Ricoeur’s philosophy of narrative identity deepens this analysis. Human beings do not inhabit time as objective chronology; time becomes human through narrative interpretation (Ricoeur 1984, 1992). Illness profoundly destabilizes this process, interrupting ordinary temporality. Palliative care therefore involves accompanying persons through disrupted temporality without reducing them to projected decline. Narrative identity remains dynamic even near the end of life: patients continue renegotiating meaning, reconstructing relationships, reinterpreting memory, and redefining hope. Algorithmic prediction intervenes directly in this narrative process by stabilizing the future through probabilistic classification. Once the patient becomes algorithmically identified as “terminal” or “high risk,” the narrative horizon may narrow, the future functioning less as open possibility and more as projected inevitability.

Contemporary medicine frequently treats uncertainty as a technical deficiency awaiting predictive correction. Yet in palliative medicine, uncertainty possesses ethical significance. It preserves existential openness, protecting the possibility that life near the end may still be narratively inhabited rather than merely administratively anticipated. Han argues that contemporary digital culture increasingly eliminates ambiguity, silence, and unpredictability through systems of continuous visibility and optimization (Han and Butler 2017). Palliative care requires the opposite ethical sensibility: to accompany a dying person is not merely to anticipate deterioration more accurately, but to preserve the existential and narrative space necessary for living meaningfully within finitude. The future may need to be anticipated; it must never become more existentially real than the person still living in the present.

## Relational compression in the algorithmic clinic

The integration of predictive AI transforms not only how prognosis is produced but also how relationships are organized within clinical care. Predictive systems alter the distribution of attention, the interpretation of vulnerability, the experience of presence, and the structure of communication between patients, families, and clinicians. This may generate *relational compression*: the progressive narrowing of narrative, symbolic, emotional, and interpersonal space within clinical encounters as predictive systems increasingly mediate attention and decision-making.

Classical palliative care is fundamentally relational: its ethical structure depends upon listening, attentiveness, embodied presence, and narrative interpretation. Predictive systems subtly modify this encounter because they increasingly shape how clinical attention is distributed. Clinicians may become oriented toward dashboards, deterioration scores, and algorithmically generated recommendations before fully encountering the patient phenomenologically. The person risks appearing first as a prognostic profile and only secondarily as a subject of lived experience. Levinas’s ethics of alterity offers an important corrective: the ethical relation begins not through classification but through exposure to the vulnerable face of the Other, who exceeds every category through which they are interpreted (Levinas 1969). No mortality score or algorithmic profile can fully contain the existential reality of suffering, dependency, memory, relational fragility, or the symbolic complexity of approaching death.

The integration of ePROMs, wearable devices, and remote monitoring intensifies this relational transformation. These tools have genuine clinical value – facilitating communication, improving symptom recognition, and strengthening patient–clinician interaction (Basch et al. 2017; Garcia Abejas et al. 2023; Greenhalgh et al. 2018). However, they simultaneously generate a new form of *anticipatory presence*: the patient remains continuously visible to healthcare systems through streams of symptom data, wearable metrics, and remote reporting infrastructures. Ordinary bodily experience becomes interpreted through digital vigilance, and the burden extends to families, who may become captured within predictive vigilance, reorganizing relationships around projected fragility (Lupton 2014). The ethical problem is not that monitoring technologies are clinically useful – they clearly may be – but that continuous visibility may inadvertently diminish existential space, transforming the patient from a person living with illness into a continuously monitored site of anticipated deterioration.

Palliative suffering frequently exceeds measurable categories. Existential distress may emerge through hesitation, silence, fragmented memory, or subtle alterations in ordinary behavior. Predictive systems inevitably struggle with such dimensions because computational logic depends upon codification and pattern recognition. What escapes datafication risks becoming less visible within digitally mediated healthcare. This is why listening remains ethically irreplaceable: to listen is to allow the patient to appear as more than a prognostic object. Relational compression occurs when measurement progressively substitutes for interpretation, when the present-moment encounter yields to anticipatory administration.

## Toward a prudential ethics of prediction

If predictive AI is to be ethically integrated into palliative medicine, the challenge is not whether prediction should exist, but how predictive knowledge ought to be situated within the moral, temporal, and relational realities of care. The ethical response cannot consist either in unconditional technological enthusiasm or in nostalgic rejection of predictive medicine. What is required is a *prudential ethics of prediction*.

Prudence, understood through the Aristotelian notion of phronesis, refers to practical wisdom exercised under conditions of fragility, uncertainty, and moral complexity (Aristotle 2009). Prudential judgment involves contextual discernment concerning what ought to be done, for whom, at what moment, under what relational conditions, and with what ethical consequences. Algorithmic systems do not eliminate the uncertainty that defines palliative care; they reorganize it. The ethical danger lies partly in the illusion that sophisticated computational systems may transform existential uncertainty into administratively manageable probability.

### Prediction without existential domination

The first principle of prudential prediction is that predictive systems must remain subordinate to lived existence rather than reorganizing lived existence around prediction. Predictive AI may estimate mortality risk, identify likely deterioration, and support referral decisions – but it must not become the dominant horizon through which the patient’s existence is interpreted. Human beings continue inhabiting illness narratively, relationally, and symbolically even under severe decline. Prediction becomes ethically problematic when it ceases to orient care and begins organizing existence.

A prudential ethics of prediction must therefore preserve *existential proportionality*: prognostic information should support preparation without prematurely occupying the totality of lived time. Patients require space not only to anticipate possible futures but to continue inhabiting the present outside continuous predictive domination – preserving uncertainty, narrative reinterpretation, ordinary continuity, silence, and forms of hope irreducible to projected outcomes. As Baxter et al. demonstrate, prognostic communication extends far beyond numerical survival estimation to encompass quality of life, relational priorities, identity, values, and the meaning patients attribute to remaining time (Baxter et al. 2024). This mediation is precisely where prudence becomes indispensable.

### Relational and narrative safeguards

The second principle concerns the protection of relational and narrative space within algorithmically mediated care. Predictive outputs should remain dialogical rather than deterministic: AI-generated risk estimates must function as prompts for interpretation and conversation, not authoritative conclusions (Ferrario et al. 2023). Uncertainty itself must remain ethically visible – predictive systems often create an impression of objective certainty through numerical precision despite remaining probabilistic, context-dependent, and temporally unstable. Clinicians must retain the interpretive freedom to contextualize predictive information within the patient’s biography, values, emotional condition, and relational world.

Narrative medicine provides an important framework here. Charon argues that clinical care requires the capacity to recognize, interpret, and accompany patient stories rather than reducing illness to biomedical abstraction (Charon 2006). Predictive AI must therefore remain situated within narrative practices capable of preserving singularity, ambiguity, and interpretive openness. This also means preserving silence and existential hesitation as legitimate dimensions of care. Human beings approaching death frequently oscillate between preparation and ordinary continuity, acceptance and denial, fear and hope. Such ambivalence is not a communicative failure requiring elimination; it belongs to the human experience of finitude.

### Reframing AI in palliative medicine

The ethical future of AI in palliative medicine depends upon how technology itself is conceptually framed. If AI is understood primarily as a system for replacing uncertainty through predictive control, palliative care risks becoming subordinated to anticipatory governance. However, predictive systems can function as relational supports rather than relational substitutes – assisting clinicians in recognizing hidden suffering, identifying unmet needs, facilitating earlier support, and overcoming prognostic paralysis (Murray et al. 2005; Neto et al. 2025). Under such conditions, AI becomes ethically valuable not because it eliminates uncertainty, but because it helps create opportunities for more humane care.

This parallels Topol’s argument that AI should augment rather than replace human medicine (Topol 2019). Within palliative care, augmentation must specifically mean relational augmentation: technologies should create more space for listening, continuity, and humane interpretation rather than reducing clinical encounters to predictive administration. A predictive system may be technically sophisticated and still ethically harmful if it compresses narrative openness, intensifies anticipatory burden, or colonizes lived time. Conversely, a system with moderate predictive accuracy may possess significant ethical value if it facilitates humane communication and preserves existential proportionality. The ethical criterion is therefore not predictive performance alone, but how AI reshapes the human experience of temporality, dignity, and care.

## Conclusion

The increasing integration of predictive artificial intelligence into palliative medicine signals a deeper transformation in how medicine understands temporality, vulnerability, and the human experience of decline. Predictive systems do not simply estimate future outcomes; they reshape how the future becomes present within clinical and existential life. In palliative medicine, where temporality possesses profound symbolic and relational density, algorithmic anticipation may alter how patients, families, and clinicians inhabit vulnerability itself.

This article introduced the notion of *algorithmic anticipatory burden* to describe the existential, relational, temporal, and institutional weight generated when AI-mediated predictions concerning deterioration or mortality become psychologically, clinically, or socially operative before the person is ready to inhabit them. When anticipated futures progressively colonize the present, narrow narrative openness, intensify anticipatory vigilance, and reorganize existence around projected decline, anticipation becomes burden.

This argument does not reject predictive medicine. Predictive systems may help overcome prognostic paralysis, facilitate earlier palliative integration, and reduce avoidable suffering. The ethical challenge is how anticipatory knowledge ought to be integrated into relational, narrative, and temporal care practices. There is a profound ethical difference between helping persons prepare for death and making them live entirely beneath its anticipation.

Against the risk of temporal colonization and relational compression, this article proposed a prudential ethics of prediction grounded in existential proportionality, narrative openness, and relational mediation. Predictive systems must remain subordinate to the ethical complexity of human finitude: supporting listening rather than compressing it, facilitating humane preparation without allowing anticipated futures to dominate the meaning of the present. Remaining time near the end of life continues to contain memory, silence, relational repair, ordinary continuity, spiritual struggle, and forms of hope that exceed probability. No predictive model can fully calculate how mortality is humanly inhabited.

The ethical future of AI in palliative medicine therefore depends upon preserving a careful balance between anticipation and humility. The patient must remain more important than the prediction. Presence must remain more important than optimization. Narrative must remain more important than classification. And lived temporality must remain irreducible to probabilistic administration. The ethical task of palliative medicine in the algorithmic age is not to abolish uncertainty through increasingly precise prediction, but to accompany human beings wisely, relationally, and compassionately within the fragility of finite existence. The future may need to be anticipated; it must never be allowed to become more powerful than the humanity of the present.

## Data Availability

No datasets were generated or analyzed during the current study.

## References

[ref1] Aristotle (2009) *The Nicomachean Ethics*. Oxford: Oxford University Press.

[ref2] Basch E, Pugh SL, Dueck AC, et al. (2017) Feasibility of patient reporting of symptomatic adverse events via the patient-reported outcomes version of the common terminology criteria for adverse events (PRO-CTCAE) in a chemoradiotherapy cooperative group multicenter clinical trial. *International Journal of Radiation Oncology*Biology*Physics* 98(2), 409–418. doi:10.1016/j.ijrobp.2017.02.002. PMC5557037 28463161

[ref3] Baxter R, Pusa S, Andersson S, et al. (2024) Core elements of serious illness conversations: An integrative systematic review. *BMJ Supportive & Palliative Care* 14(e3), e2268. 10.1136/spcare-2023-004163 PMC11671901 37369576

[ref4] Charon R (2006) *Narrative Medicine: Honoring the Stories of Illness*. Oxford University Press.

[ref5] De Panfilis L, Peruselli C, Tanzi S, et al. (2023) AI-based clinical decision-making systems in palliative medicine: ethical challenges. *BMJ Supportive & Palliative Care* 13(2), 183–189. 10.1136/bmjspcare-2021-002948 34257065

[ref6] Ferrario A, Gloeckler S and Biller-Andorno N (2023) Ethics of the algorithmic prediction of goal of care preferences: From theory to practice. *Journal of Medical Ethics* 49(3), 165. 10.1136/jme-2022-108371 PMC9985740 36347603

[ref7] Garcia Abejas A, Serra Trullas A, Sobral MA, et al. (2023) Improving the understanding and managing of the quality of life of patients with lung cancer with electronic patient-reported outcome measures: Scoping Review. *Journal of Medical Internet Research* 25, e46259. 10.2196/46259 PMC10193214 37021695

[ref8] Greenhalgh J, Gooding K, Gibbons E, et al. (2018) How do patient reported outcome measures (PROMs) support clinician-patient communication and patient care? A realist synthesis. *Journal of Patient-Reported Outcomes* 2, 42. 10.1186/s41687-018-0061-6 PMC6153194 30294712

[ref9] Han BC and Butler E (2017) *Psychopolitics: Neoliberalism and New Technologies of Power*. Verso Books.

[ref10] He JC, Moffat GT, Podolsky S, et al. (2024) Machine learning to allocate palliative care consultations during cancer treatment. *Journal of Clinical Oncology* 42(14), 1625–1634. 10.1200/JCO.23.01291 38359380

[ref11] Heidegger M (1996) *Being and Time: A Translation of Sein Und Zeit*. State University of New York Press.

[ref12] Heinzen EP, Wilson PM, Storlie CB, et al. (2023) Impact of a machine learning algorithm on time to palliative care in a primary care population: Protocol for a stepped-wedge pragmatic randomized trial. *BMC Palliative Care* 22(1), 9. 10.1186/s12904-022-01113-0 PMC9896817 36737744

[ref13] Levinas E (1969) *Totality and Infinity: An Essay on Exteriority*. Duquesne University Press.

[ref14] Liu JH, Shih CY, Huang HL, et al. (2023) Evaluating the potential of machine learning and wearable devices in end-of-life care in predicting 7-day death events among patients with terminal cancer: Cohort study. *Journal of Medical Internet Research* 25, e47366. 10.2196/47366 PMC10474512 37594793

[ref15] Lupton D (2014) Self-tracking cultures: Towards a sociology of personal informatics. In *Proceedings of the 26th Australian Computer-Human Interaction Conference on Designing Futures: The Future of Design*, Sydney, New South Wales, Australia. doi:10.1145/2686612.2686623.

[ref16] Murray SA, Boyd K and Sheikh A (2005) Palliative care in chronic illness. *BMJ* 330(7492), 611–612. 10.1136/bmj.330.7492.611 PMC554893 15774965

[ref17] Neto IG, Sarmento T and Bruera E (2025) Breaking free from prognostic paralysis in chronic advanced diseases. *Palliative and Supportive Care* 23, e124. 10.1017/S1478951525100254 PMC13166679 40545622

[ref18] Porter AS, Harman S and Lakin JR (2020) Power and perils of prediction in palliative care. *The Lancet* 395(10225), 680–681. 10.1016/S0140-6736(20)30318-4 32113499

[ref19] Ricoeur P (1984) *Time and Narrative*, Vol 2, University of Chicago Press.

[ref20] Ricoeur P (1992) *Oneself as Another*. University of Chicago Press.

[ref21] Strand JJ, Morgan AA, Pachman DR, et al. (2024) Performance of an artificial intelligence/machine learning model designed to identify hospitalized patients with cancer who could benefit from timely specialized palliative care delivery. *Journal of Clinical Oncology* 42(16_suppl), 1559–1559.

[ref22] Topol E (2019) *Deep Medicine: How Artificial Intelligence Can Make Healthcare Human Again*. Basic Books.

[ref23] Wilson PM, Ramar P, Philpot LM, et al. (2023) Effect of an artificial intelligence decision support tool on palliative care referral in hospitalized patients: A randomized clinical trial. *Journal of Pain and Symptom Management* 66(1), 24–32. 10.1016/j.jpainsymman.2023.02.317 36842541

